# The Persistent Circulation of Enterovirus 71 in People's Republic of China: Causing Emerging Nationwide Epidemics Since 2008

**DOI:** 10.1371/journal.pone.0025662

**Published:** 2011-09-28

**Authors:** Xiaojuan Tan, Xueyong Huang, Shuangli Zhu, Hui Chen, Qiuli Yu, Haiyan Wang, Xixiang Huo, Jianhui Zhou, Yan Wu, Dongmei Yan, Yong Zhang, Dongyan Wang, Aili Cui, Hongqiu An, Wenbo Xu

**Affiliations:** 1 National Institute for Viral Disease Control and Prevention, Chinese Center for Disease Control and Prevention, Beijing, China; 2 Henan Provincial Center for Disease Control and Prevention, Zhengzhou, Henan, China; 3 Ningxia Provincial Center for Disease Control and Prevention, Yinchuan, Ningxia, China; 4 Hebei Provincial Center for Disease Control and Prevention, Shijiazhuang, Hebei, China; 5 Shandong Provincial Center for Disease Control and Prevention, Jinan, Shandong, China; 6 Hubei Provincial Center for Disease Control and Prevention, Wuhan, Hubei, China; 7 Jilin Provincial Center for Disease Control and Prevention, Changchun, Jilin, China; 8 Yunnan Provincial Center for Disease Control and Prevention, Kunming, Yunnan, China; University of Rochester, United States of America

## Abstract

Emerging epidemics of hand-foot-and-mouth disease (HFMD) associated with enterovirus 71 (EV71) has become a serious concern in mainland China. It caused 126 and 353 fatalities in 2008 and 2009, respectively. The epidemiologic and pathogenic data of the outbreak collected from national laboratory network and notifiable disease surveillance system. To understand the virological evolution of this emerging outbreak, 326 VP1 gene sequences of EV71 detected in China from 1987 to 2009 were collected for genetic analyses. Evidence from both traditional and molecular epidemiology confirmed that the recent HFMD outbreak was an emerging one caused by EV71 of subgenotype C4. This emerging HFMD outbreak is associated with EV71 of subgenotype C4, circulating persistently in mainland China since 1998, but not attributed to the importation of new genotype. Originating from 1992, subgenotype C4 has been the predominant genotype since 1998 in mainland China, with an evolutionary rate of 4.6∼4.8×10^−3^ nucleotide substitutions/site/year. The phylogenetic analysis revealed that the majority of the virus during this epidemic was the most recent descendant of subgenotype C4 (clade C4a). It suggests that the evolution might be one of the potential reasons for this *native* virus to cause the emerging outbreak in China. However, strong negative selective pressure on VP1 protein of EV71 suggested that immune escape might not be the evolving strategy of EV71, predicting a light future for vaccine development. Nonetheless, long-term antigenic and genetic surveillance is still necessary for further understanding.

## Introduction

Enterovirus 71 (EV71) is a member of human enterovirus group A (HEV-A) species, belongs to *Enterovirus* genus in *Picornaviridae* family. Since it was first identified from a child with neurologic symptoms in California in 1969 [1], outbreaks associated with EV71 have been reported worldwide [2]–[7]. During late 20^th^ century, several large outbreaks of hand-foot-and-mouth disease (HFMD) associated with EV71 infections have been reported in Eastern and Southeastern Asian countries and regions, e.g., Malaysia in 1997 [8], Singapore in 1998 [9], and Taiwan in 1998 [10]. The large scale and a number of fatal cases made these outbreaks distinguishing and attract the global attentions. Since then, outbreaks of EV71 frequently occurred in Asian countries, with high incidence of neurologic infection and fatality [8], [11]–[14].

In China, large scale outbreak of HFMD associated with EV71 emerged in 2007. which occurred in Linyi City, Shandong Province, with 1,149 mild and 3 fatal cases reported [15]. The nationwide epidemics of EV71 started in 2008, beginning as an outbreak of unknown viral infection in Anhui province, and spreading into other provinces quickly [16]–[18]. Totally 488,955 children were suffered from this epidemic and 126 lives were claimed due to neurogenic cardiopulmonary failure and brain stem encephalitis [18], [19]. To respond to the epidemic, a national HFMD surveillance system was established in May 2008 so an effective prevention strategy can be implemented. In 2009, the epidemics of EV71 continued and became much more rampant.

VP1 gene is of major significance for virological surveillance of enterovirus because it contains a number of importance neutralization epitopes [20], [21]. In this study, we performed genetic analyses on VP1 sequences using isolates detected in mainland China from 1987 to 2009 to investigate the evolution and molecular epidemiology of EV71 in this country. Although there are already a few previous reports about evolutionary analysis on global EV71 [22], [23], the data on subgenotype C4 especially from China after 2007 were little included. Through the analyses of sequence data from mainland China, we aimed to find some clues to explain the sudden outbreak of EV71 infections in China, and provide useful advice from virology for the disease control in the future.

## Results

### 1. Surveillance of HFMD in China

To collect epidemiologic information and understand the recent epidemics, HFMD was listed as a Class C notifiable disease in the national surveillance system on May 2, 2008. It requires the information of every case be submitted to the National Notifiable Disease Reporting System (NNDRS) within 24-hour upon diagnosed.

The National Laboratory Network (excluding Hong Kong, Macau, and Taiwan) for HFMD was organized in 2008 to incorporate 331 prefectural laboratories at prefectural Centers for Disease Control and Prevention (CDCs), 31 provincial laboratories at provincial CDCs, and 1 national laboratory at China CDC. Collection of patient information and specimen followed the guidelines in the *Manual for Hand, Foot, and Mouth Disease control and prevention* released by the Ministry of Health (www.moh.gov.cn/publicfiles/business/htmlfiles/mohbgt/s3582/200906/41047.htm). Prefectural laboratory conducts tests using RT-PCR or real time RT-PCR on clinical samples to identify the infections of EV71, CA16, and other enterovirus. Provincial laboratory performs virus isolations and serotyping on positive samples from prefectural laboratory. The identified EV71 and CA16 isolates are forwarded to National laboratory for sequencing and molecular analysis.

A total of 488,955 HFMD cases including 1,165 severe and 126 fatal cases were reported to NNDRS in 2008 [18]. In 2009, the outbreak continued. There was an apparently increase of the reported national incidence rate of HFMD, 87 hundred-thousandth in 2009 to 37 hundred-thousandth in 2008. Totally 1,166,131 cases with 13,810 severe cases and 353 fatalities were reported throughout 2009. Children younger than 5 years old were the majority of the victims in the outbreaks, which accounted for 91.3% (446,263 cases) of the reported cases in 2008, and 93.2% (1,086,793 cases) in 2009, respectively.

Laboratory results revealed that EV71 and CA16 were the major pathogens for the recent HFMD outbreaks. In 2008, 54.4% and 14.6% of the cases confirmed were associated with EV71 and CA16 infections, respectively. In 2009, EV71 and CA16 were still the major pathogens, responsible for 48.1% and 31.5% of the confirmed cases, respectively. However, EV71 infections were predominant for severe and fatal cases which caused 81.6% of the severe cases and 95.3% of the fatalities in 2008, and 80.6% and 92.8% accordingly in 2009.

### 2. Molecular epidemiology of EV71 in China

The complete VP1 gene sequence data of the available EV71 strains isolated in China were used for this study, including released sequences from GenBank before February 23^rd^, 2010 and sequences from our laboratory. In China, the first EV71 strain was isolated from an adult HFMD case in 1987 [24]. Successive molecular virologic data on EV71 was available since 1998. After the establishment of laboratory network for HFMD in 2008, a large amount of data on etiology and molecular epidemiology become available, Strains isolated from 18 provinces during 1987-2009 were included, 87% (283/326) of the sequences data collected were between 2007 and 2009 (Table S3).

**Figure 1:**
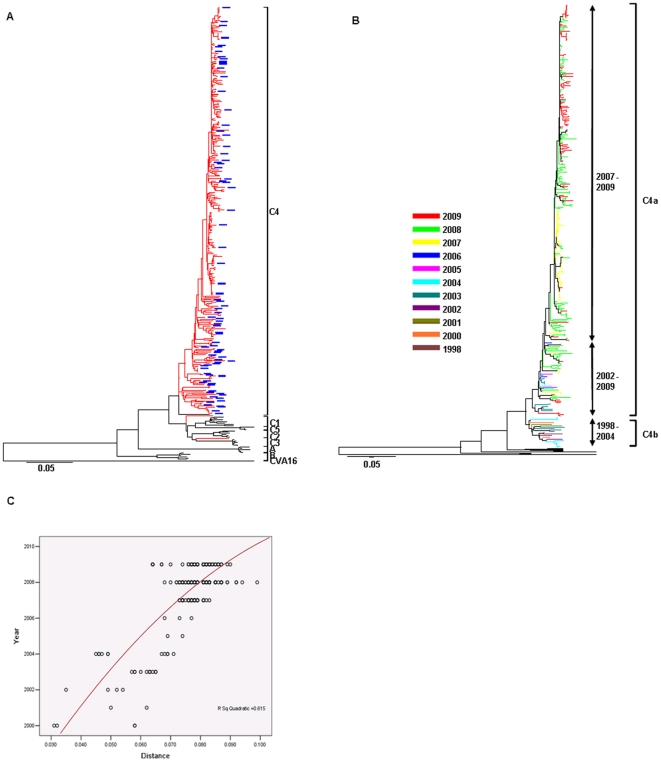
Phylogenetic analyses based on all the available VP1 Nt sequences of EV71 in China. (**A**) Phylogenetic tree showed that subgenotype C4 was the predominant one in mainland China. The branches of sequences from China were highlighted in red color. The sequences marked with blue block were selected and used for Baysian MCMC computations and natural selection inference. (**B**) Phylogenetic tree showed the evolutionary tendency of subgenotype C4 strains in China since 1998. The branches were colored differently by years. A ladder-like topology revealed the adaptation of EV71 occurred during the epidemic history [32], [33]. (**C**) Illustration of genetic evolution over years for all the strains of subgenotype C4 in China with distance measured to SHZH98 (AF302996; the first C4 strain isolated in China mainland). The distances conformed to tree distance in (A) and (B). The red line is the best-fit regression curve fitted all the data point to indicate the trend through time.

In our study, a total of 326 complete VP1 genome sequences of EV71 isolates from mainland China were collected (Table S1). Phylogenetic dendrograms were constructed based on these complete VP1 gene sequences (Figure 1). It was shown that all the EV71 circulating in China since 1998 were clustered with strains of subgenotype C4, except for 0667-CHN-87 (isolated in 1987, belonged to genotype C but not any subgenotype) and 97-56-CHN-97 (isolated in 1997, belonged to subgenotype C3) (Fig. 1A). The first strain of subgenotype C4 (SHZH98) documented in mainland China was detected in 1998 in Guangzhou [25]. Since 1998, the predominant subgenotype C4 has been circulating in China for at least 11 years, the epidemic pattern of which was very different from other countries and regions. In other neighboring countries and regions, EV71 of several subgenotypes circulated simultaneously during the outbreaks, and in some regions subgenotype transitions happened for several times throughout the period [14], [26]–[29]. For example, in Taiwan, C2 was the major subgenotype co-circulated with B4 during the epidemics in 1998; in the subsequent outbreaks in 2000, B4 became a dominant subgenotype; then C4 in 2004, and B5 and C5 in 2006 [30], [31]. Although mainland China is neighboring to Taiwan, subgenotype C4 was the unique type detected in China since 1998, during which there was no transition or co-circulation of any other genotypes (Fig. 1A). This characteristic made the successive data of EV71 of subgenotype C4 in China invaluable for understanding the evolution of EV71.

The sequences of C4 were analyzed further. All sequences were grouped by the year of sampling and showed with different colors in the phylogenetic tree (Figure 1B). It was revealed that all the EV71 of subgenotype C4 from China could be split into two clades (Fig 1B), which were designated as C4a and C4b, respectively [15]. The strains of C4b were detected only before 2004, and C4a included all the isolates since 2005 and some isolates from 2002 to 2004. Briefly, subgenotype C4 had displayed 3 major evolutionary phases in China (Figure 1B): the first phase, from 1998 to 2001, in which all isolates belonged to clade C4b; in second phase, during clade transition, from 2002 to 2004, isolates of both clade C4b and C4a co-circulated; in third phase, after 2005, only C4a were detected. The clade transition suggested that evolution of EV71 of subgenotype C4 occurred during persistent circulation in mainland China within 11 years. The genetic progression (Figure 1C) showed that the genetic distance to SHZH98 (AF302996), the first isolate of subgenotype C4 in China, became larger over time, which also suggested the evolution of subgenotype C4 in China.

The strains detected during the recent outbreaks, from 2007 to 2009, interspersed in clade C4a with the isolates before 2007, but most of them clustered with each other and located at the terminal of the phylogenetic tree (Fig 1B), which meant that the most recent descendant from C4a was responsible mainly for the current large outbreaks (Fig 1B and 1C). It suggested that evolution might play some roles in recent epidemics of EV71 infections [32], [33].

### 3. The evolution of subgenotype C4 in China

#### Origin and evolutionary rate

To study the evolutionary characteristics of subgenotype C4 in China, 82 VP1 sequences of subgenotype C4 (Table S1), sampled from 1998 to 2009, were selected for divergence time and substitution rate estimation with Bayesian Markov chain Monte Carlo (MCMC) method [25], [34], [35].

The 82 sequences selected for evolutionary analysis (detailed in methods) were all typical representatives in phylogenetic tree, which was labeled with blue-block in Figure 1A. Totally 8 different models were used for data analyses (Table 1). It was found that the GTR and uncorrelated lognormal distribution clock model (GTR+UCLD) fitted to our data best. The coefficient of variation of the evolutionary rates among branches in the UCLD model was 0.358 (95% highest posterior density [HPD]: 0.176–0.550), significantly different from 0, which indicated that rates heterogeneity existed among different branches and the evolution of EV71 was not clock-like in China also, consistent with results from global data [25].

**Table 1:** Summary of 8 different models for evolutionary analyses.

	UCLD				strict clock			
	GTR+CS	GTR+EG	HKY+CS	HKY+EG	GTR+CS	GTR+EG	HKY+CS	HKY+EG
Marginal Likelihood	−5584.36	−5585.14	−5613.54	−5613.97	−5603.71	−5603.38	−5631.56	−5631.08
t_MRCA_	15.8(13.4–18.3)	15.1(13.0–17.4)	15.8(13.6–18.4)	15.1(13–17.3)	16.1(14.4–17.9)	15.8(14.1–17.6)	16.0(14.3–17.8)	15.7(14.1–17.5)

Note: The marginal likelihood and t_MRCA_ in the 8 different models were showed in this table. The improvement in marginal likelihood suggested that the GTR substitution model was superior to the HKY model [35]. And the relaxed molecular clock model of uncorrelated lognormal-distributed (UCLD) rate variation among branches fit our data better than strict clock model [34]. The constant (CS) and exponential growth (EG) population size had no significant impact on the analyses. The t_MRCA_ was in units of years before 2009, the 95% HPD was showed in the parentheses.

According to our analyses, the most recent common ancestor of subgenotype C4 strains in China can be traced to 15.1–16.1 (95% HPD: 13.0–18.4) years before 2009 (Table 1). The divergence time was deduced to be from Oct. 1992 to Oct. 1993 (95% HPD: Jul. 1990–Jan. 1996), approximately equal to the global subgenotype C4 divergence time estimated in previous study [23]. This evidence suggested that subgenotype C4 strain might begin to circulate in mainland China immediately as it diverged as a novel subgenotype from other genotypes. And EV71 of subgenotype C4 had been circulating for about 5–6 years in China before it was first detected in 1998, consistent to a previous report which estimated that genogroup B and C had each circulated cryptically in the population for up to 5 years before causing large HFMD outbreaks [23].

Subgenotype C4 evolved in China at inconstant rate, and the estimated mean evolutionary rate among different branches was 4.6×10^−3^ and 4.8×10^−3^ substitutions per site per year inferred by the models of GTR+CS+UCLD and GTR+EG+UCLD, respectively (Table 1), which was approximate to estimates of global genotype B and C estimated also by MCMC method previously [23].

#### Epidemic history referred by molecular sequence

**Figure 2:**
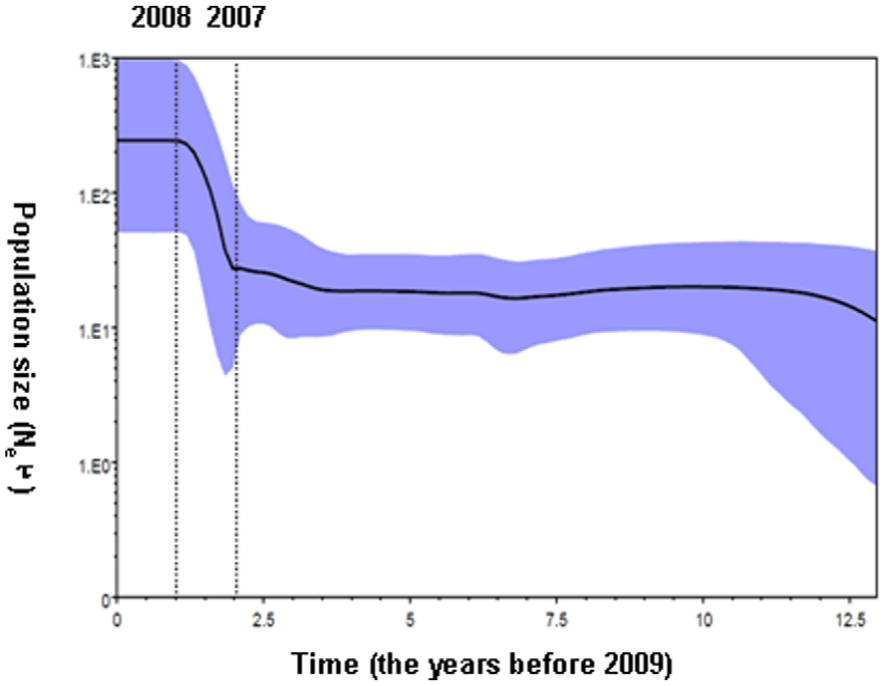
Baysian skyline plot was estimated to reconstruct the demographic history of subgenotype C4 EV71 in China [**36**]. The x axis is in units of years before 2009, and the y axis is equal to N_e_τ (the product of the effective population size and the generation length in years). The thick solid line is the mean estimates, the 95% HPD credible region is showed by blue areas.

To exclude that the recent outbreak of HFMD is an artifact of increased reporting attributed to the establishment of NNDRS in 2003, the demographic history of subgenotype C4 in China was reconstructed with Bayesian skyline plot method based on the VP1 sequences data [36]. The plot showed a sharp increase (Figure 2), indicating an exponential growth of the population size (reflected the effective infections) occurred from 2007 to 2008, which was consistent with the epidemiological data about the large-scale HFMD outbreak in 2008. This finding revealed that the outbreak of EV71 in 2008 was actually ascribed to the explosion of viral population but not the improved surveillance sensitivity.

#### Natural selection analysis

We conducted natural selection analyses with SLAC and FEL method respectively on the Datamonkey website service. The results showed a low mean *dN/dS* ratios (0.055) of subgenotype C4 in VP1 (297 codons). Totally 127 codons and 202 codons (details was not shown) were identified as negatively selected sites by SLAC and FEL respectively, and only the codon 98 of VP1 protein was under positive selection pressure (P-value <0.1). The results suggested that VP1 protein was under strongly negative selection pressure. Because of the importance of VP1 protein on neutralizing epitopes [20], [21], negative selection pressure on VP1 meant that variations of VP1 protein weren't motivated by selection pressure from host immune system but limited by its important functions [33]. In other words, the variation of VP1 sequence was mainly ascribed to the lack of correcting function during the genome replication of RNA virus, and the deleterious mutations were cleared [37]. The evolution of VP1 protein was mainly due to the accumulation of random mutations during genome replication; a small portion of non-synonymous mutations induced amino acid changes but not directed variations.

## Discussion

HFMD epidemic has become a serious public health concern in China since 2007. Laboratory and epidemiology surveillance system of HFMD had been established in mainland China in 2008. Virologic data from laboratory network revealed that EV71 infections were the main reason for severe and fatal cases, although both EV71 and Coxsackievirus A16 were the major pathogens during the widespread outbreaks in China from 2008 to 2009.

Before 2008, only several small outbreaks with mild cases were documented and no compulsory surveillance on HFMD in China. In our study, both the epidemiology and epidemic history referred by molecular sequences (Figure 2) support the recently sudden outbreak of EV71 infections as an emerging one in China but not the artifact of more sensitive reporting.

The emergence and reemergence of infectious disease could be attributed to either accumulated susceptible population or appearance of pathogens with new biological characteristics. For EV71 infections, children less than 5 years old are the majority of susceptible group. Our retrospective seroepidemiology study reported in previous study [38] revealed that the circulation of EV71 had been established widely in China before 2005. Our unpublished study (discussed in reference 39) on EV71-seroprevalence rates among young children aged 1-5 years in Fuyang during and after the outbreak in 2008 [17] showed that the post-epidemic seroprevalence rate had a slight increase. The seroepidemiologic results in China, which are consistent with previous studies from Taiwan [10], [39], suggested that susceptible populations may play some role in the nationwide epidemics, but might not be the major reason.

This study performed molecular epidemiologic and evolutionary analyses on EV71 detected in China through 2009, trying to find some explanations for the recent outbreaks from the virology point of view. The results indicated that EV71 genotype C4 was the unique subgenotype detected in mainland China since 1998 (Figure 1A), which originated from 1992 to 1993 and has been circulating persistently for more than 12 years. But it was associated with nationwide HFMD outbreak in last three years, and causing quite a few severe and fatal cases. Evidence above suggested that the recent outbreak in China was an emerging one caused by the *native* virus but not the importation of new genotype. Differently to mainland China, emergence and reemergence of EV71 outbreaks in other countries and regions were associated with establishment of the new genotype [23], [30].

According to our phylogenetic analyses on VP1 sequences, in China evolution and clade transition of subgenotype C4 occurred during the circulation of more than 12 years, and the most recent descendants of subgenotype C4 were the majority EV71 strains detected during the outbreaks since 2007 (Figures 1B and 1C). It suggested that the evolution of EV71 strains might be responsible for the recent HFMD outbreak which associated with subgenotype C4 virus. After the long and persistent circulation, the recent EV71 strains in China might have gained new characteristics making it more aggressive, such as stronger transmissibility, infectivity and (or) virulence. But the mechanism is still unclear because of the lack of suitable animal model, advanced researches are still needed. Fortunately, the recent discovery of cellular receptors of EV71 provides us better understanding on the binding and infection of EV71, also presents promising opportunity to develop transgenic animal model of EV71 [40]-[42].

For enterovirus control, e.g., polio eradication, the trivalent oral poliovirus vaccine (tOPV) is the only one using in China currently. For prevention and control of HFMD, vaccine is probably the most effective method to fight against viral infections. Inactivated EV71 vaccine would be the most hopeful so far, attributing to the simpler technology and higher safety. However, whether the incipient vaccine strains could maintain effective for long term like polio vaccine or the strains should be optimized periodically according to surveillance as in the case of influenza virus? So far, Limited information on antigenic evolution of EV71 is available. Our estimated mean evolutionary rate of EV71 was ∼ 4.6×10^−3^ substitutions/site/year, which is approximate to that of the influenza [43] and much less frequently than polio virus (1.03×10^−2^) [44], the typical species of enterovirus with antigenic stable for long [45]. However, results on natural selection revealed that VP1 protein, the most important region of antigenic epitopes [46], of subgenotype C4 in China was under strongly negative selection pressure, similar with that of polio [45] but influenza [43] (which is also discussed in reference [23]). Moreover, various subgenotypes could be neutralized by the antibodies induced after EV71 infection although several subgenotypes of EV71 have emerged [11], [26], [47] since first identified in 1963 attributed to the genomic evolution [30], [48], [49], indicating that the antigenicity of EV71 still keep stable during evolution. Accordingly, it was speculated that the antigenicity of EV71 in China would keep stable for a long period like polio. This characteristic would make the development of EV71 vaccine much simpler, which means that it is not necessary to optimize the vaccine strains periodically due to the antigenic variation. However, still a few codons (only codon 98 in this study) of VP1 were demonstrated to be under positive selection by both this and previous studies [22], [23], [30]. Besides the selective pressure on VP1 codons, potential recombination could also induce antigenic variation in EV71. Additionally, there are still 3 other capsid proteins (VP2-VP4) presence. The antigenic evolutionary characteristics of EV71 was not fully comprehended as described by a previous research [30]. Therefore, a long term antigenic surveillance for EV71 is necessary. The persistent circulation of subgenotype C4 in China made our data invaluable for exploring the evolutionary characteristics of EV71, which should remain as the priority in our future researches.

Because of its wide territory and large population in mainland China, the epidemics of EV71 might still need another several years to sweep out the susceptible population. Additionally, the new born population which refresh the susceptible pool annually and the nonsynchronous epidemics of EV71 among different provinces also determine that the current epidemic is still going to continue in the near future. The epidemics of EV71 infections would still harass China for some time as prediction, and a harsh combat in control and prevention is yet to come.

## Materials and Methods

### 1. Virus isolation and identification

After processing, specimens from clinically diagnosed HFMD patients were inoculated into appropriate cell cultures including HEp-2, RD, and Vero cells. EV71 strains were identified by RT-PCR and sequencing of VP1 genome (891 nucleotides).

### 2. Sequencing and sequence collection of EV71

RT-PCR amplification and sequencing of VP1 encoding gene were performed as previously described [15]. Thirty-one sequences have been deposited in GenBank (accession number HM212436- HM212466). Other EV71 strains with complete VP1 sequences isolated from mainland China were downloaded from GenBank which were released before February 23^rd^, 2010. The sequence information was checked manually to exclude the laboratory adaptive strains, clones, and strains with high passage numbers. Eventually 295 complete VP1 gene sequences with known collection dates between 1987 and 2009 were obtained. Totally, 326 EV71 isolates were included in this study as shown in Table S1.

### 3. Phylogenetic analyses

For phylogenetic analysis, reference strains of A, B, and C1-C5 genotype/subgenotype [11], [47] were obtained from GenBank as listed in Table S2. Sequence alignment of Chinese and reference strains was conducted with ClustalW program in MEGA 4 (Molecular Evolutionary Genetics Analysis software; Tamura, Dudley, Nei, and Kumar 2007). A phylogenetic tree based on complete VP1 coding sequence was constructed with Kimura 2-Parameter model and Neighbor-Joining method, using MEGA. The bootstrap test was performed with 1,000 replications.

### 4. Evolutionary analyses based on Bayesian MCMC method

The evolution rate, time of most recently common ancestor (t_MRCA_), molecular clock phylogeny, and demographic history of the EV71 strains circulating in mainland China were inferred using Bayesian Markov chain Monte Carlo (MCMC) method in BEAST version 1.4.8 (www.beast.bio.ed.ac.uk). Because of the large epidemic of EV71 in China since 2007, a great amount of the sequence data were collected from 2007 to 2009, to reducing the computation load, sequences with high homogeneity (e.g., from the same outbreak) were deleted. Thus, 82 representative sequences were selected for the evolutionary and natural selection analyses. We analyzed the data using both the Hasegawa-Kishino-Yano (HKY) and the General Time Reversible (GTR) Nt substitution models with the gamma distribution of among-site rate variation with four categories estimated from the empirical data. The codon positions were partitioned into 1^st^ +2^nd^ and 3^rd^, in which 3^rd^ codon position had a separate relative substitution rate parameter with others. Also, two different models of rates variation among branches: the strict clock and the uncorrelated lognormal distributed (UCLD) relaxed molecular clock were implemented in our analyses [25], [34]. Both constant and exponential growth population size coalescent were used as tree prior to avoid impact from different demographic models. For each model the MCMC chain was run for 30,000,000 steps and sampled every 1,000 steps. The first 3,000,000 steps of each run were discarded as burn-in. This ensured that the effective sample sizes for all the parameters were more than 500 for all the models. To reconstruct the demographic history of EV71 in China, Baysian skyline plot was estimated [36], inferring the tendency of effective infections since t_MRCA_ in China.

### 5. Natural selection analyses

The nonsynonymous and synonymous substitution rates (dN and dS, respectively), and the *d*N/*d*S ratio were computed to estimate the natural selection pressure on each VP1 codon of EV71. Both Single-likelihood ancestor counting (SLAC) and fixed-effects likelihood (FEL) methods at the Datamonkey website (www.datamonkey.org) were performed. In SLAC method, if dN/dS>1 (or <1), a codon was called positively (or negatively) selected, p-value (0.1) derived from a two-tailed extended binomial distribution was used to assess significance. P-value (0.1) from one degree of freedom likelihood ratio test was used in FEL method to assess whether the site was under positive or negative selection.

### 6. Ethics Statement

HFMD is a notifiable disease in China, and the pathogenic surveillance of HFMD without private information referred is required by the Law of the PRC on the Prevention and Treatment of Infectious Diseases. The data used in this study was obtained from samples as part of this monitoring program and according to this law. No identifying data were used in this study. Thus, the requirement for written informed consent was waived. This study was approved by the second session of Ethics Review Committee in Chinese Centre for Disease Control and Prevention.

## Supporting Information

Table S1 The list of EV71 strains from China used for analysis in this study.
(DOC)

Table S2 Reference strains used for phylogenetic analysis (CA16 was used as out-group control).
(DOC)

Table S3 The locality and time distribution of the EV71 VP1 sequences involved in this study.
(DOC)
