# Supplementary material for: The Persistent Circulation of Enterovirus 71 in People's Republic of China: Causing Emerging Nationwide Epidemics Since 2008
Source: PLoS One. 2011 Sep 28;6(9):e25662. doi: 10.1371/journal.pone.0025662 (PMC3181342; doi:10.1371/journal.pone.0025662)
Supplement: Table S1 — The list of EV71 strains from China used for analysis in this study. (DOC) [file pone.0025662.s001.doc]

| Isolated years | Provinces | Strains name | GenBank accession No. | Used for | Origin |
| --- | --- | --- | --- | --- | --- |
| 1987 | Hubei | 0667-CHN-87 | AF135934 | Phylogenetic analysis | GenBank |
| 1997 | Heilongjiang | 97-56-CHN-97 | AB115494 | Phylogenetic analysis | GenBank |
| 1998 | Guangdong | SHZH98 | AF302996 | Phylogenetic & Evolutionary analysis | GenBank |
| 2000 | Shanghai | F1-CHN-00 | AB115490 | Phylogenetic analysis | GenBank |
| 2000 | Shanghai | F2-CHN-00 | AB115491 | Phylogenetic & Evolutionary analysis | GenBank |
| 2000 | Shanghai | H25-CHN-00 | AB115492 | Phylogenetic analysis | GenBank |
| 2000 | Shanghai | H26-CHN-00 | AB115493 | Phylogenetic & Evolutionary analysis | GenBank |
| 2001 | Guangdong | shzh01-3 | AY895132 | Phylogenetic & Evolutionary analysis | GenBank |
| 2001 | Guangdong | shzh01-4 | AY895134 | Phylogenetic & Evolutionary analysis | GenBank |
| 2002 | Guangdong | shzh02-40 | AY895130 | Phylogenetic & Evolutionary analysis | GenBank |
| 2002 | Guangdong | shzh02-62 | AY895136 | Phylogenetic & Evolutionary analysis | GenBank |
| 2002 | Shanghai | EV71-SHH02-17 | AY547500 | Phylogenetic & Evolutionary analysis | GenBank |
| 2002 | Shanghai | EV71-SHH02-6 | AY547499 | Phylogenetic & Evolutionary analysis | GenBank |
| 2003 | Chongqing | EV71-CQ03-1 | AY547501 | Phylogenetic & Evolutionary analysis | GenBank |
| 2003 | Guangdong | SHZH03 | AY465356 | Phylogenetic & Evolutionary analysis | GenBank |
| 2003 | Guangdong | shzh03-105 | AY895133 | Phylogenetic & Evolutionary analysis | GenBank |
| 2003 | Guangdong | shzh03-106 | AY895138 | Phylogenetic & Evolutionary analysis | GenBank |
| 2003 | Guangdong | shzh03-58 | AY895135 | Phylogenetic & Evolutionary analysis | GenBank |
| 2003 | Shandong | TS011/SD/CHN/2003 | HM212441 | Phylogenetic & Evolutionary analysis | this study |
| 2003 | Shandong | TS013/SD/CHN/2003 | HM212442 | Phylogenetic & Evolutionary analysis | this study |
| 2003 | Shandong | TS014/SD/CHN/2003 | HM212443 | Phylogenetic & Evolutionary analysis | this study |
| 2003 | Zhejiang | ZJ-CHN-1-03 | AY905614 | Phylogenetic & Evolutionary analysis | GenBank |
| 2003 | Zhejiang | ZJ-CHN-2-03 | AY905615 | Phylogenetic & Evolutionary analysis | GenBank |
| 2003 | Zhejiang | ZJ-CHN-3-03 | AY905616 | Phylogenetic analysis | GenBank |
| 2003 | Zhejiang | ZJ-CHN-4-03 | AY905617 | Phylogenetic analysis | GenBank |
| 2003 | Zhejiang | ZJ-CHN-5-03 | AY905618 | Phylogenetic analysis | GenBank |
| 2003 | Zhejiang | ZJ-CHN-6-03 | AY905619 | Phylogenetic analysis | GenBank |
| 2004 | Guangdong | shzh04-12 | AY895144 | Phylogenetic & Evolutionary analysis | GenBank |
| 2004 | Guangdong | shzh04-2 | AY895139 | Phylogenetic analysis | GenBank |
| 2004 | Guangdong | shzh04-20 | AY895143 | Phylogenetic & Evolutionary analysis | GenBank |
| 2004 | Guangdong | shzh04-23 | AY895141 | Phylogenetic analysis | GenBank |
| 2004 | Guangdong | shzh04-3 | AY895142 | Phylogenetic analysis | GenBank |
| 2004 | Guangdong | shzh04-38 | AY895145 | Phylogenetic analysis | GenBank |
| 2004 | Guangdong | shzh04-J38 | AY895129 | Phylogenetic analysis | GenBank |
| 2004 | Guangdong | shzh04-J39 | AY895140 | Phylogenetic analysis | GenBank |
| 2004 | Guangdong | shzh04-J40 | AY895137 | Phylogenetic & Evolutionary analysis | GenBank |
| 2004 | Guangdong | shzh04-J41 | AY895092 | Phylogenetic analysis | GenBank |
| 2004 | Guangdong | shzh04-J42 | AY895131 | Phylogenetic & Evolutionary analysis | GenBank |
| 2005 | Shandong | 05273/SD/CHN/2005 | GQ253420 | Phylogenetic & Evolutionary analysis | GenBank |
| 2005 | Shandong | 05488/SD/CHN/2005 | GQ253421 | Phylogenetic & Evolutionary analysis | GenBank |
| 2006 | Anhui | AnHui-HeFei-1 | EU697903 | Phylogenetic & Evolutionary analysis | GenBank |
| 2006 | Shandong | 06272/SD/CHN/2006 | GQ253422 | Phylogenetic & Evolutionary analysis | GenBank |
| 2006 | Shandong | 06282/SD/CHN/2006 | GQ253423 | Phylogenetic & Evolutionary analysis | GenBank |
| 2007 | Beijing | BJ4211 | EU024958 | Phylogenetic & Evolutionary analysis | GenBank |
| 2007 | Beijing | BJ4243 | EU019910 | Phylogenetic & Evolutionary analysis | GenBank |
| 2007 | Inner Mongolia | 0708T/NM/CHN/07 | EU910861 | Phylogenetic analysis | GenBank |
| 2007 | Inner Mongolia | 0709F/NM/CHN/07 | EU910862 | Phylogenetic analysis | GenBank |
| 2007 | Inner Mongolia | 0711F/NM/CHN/07 | EU910863 | Phylogenetic analysis | GenBank |
| 2007 | Inner Mongolia | 0712F/NM/CHN/07 | EU910864 | Phylogenetic analysis | GenBank |
| 2007 | Inner Mongolia | 0715F/NM/CHN/07 | EU910865 | Phylogenetic analysis | GenBank |
| 2007 | Inner Mongolia | 0716F/NM/CHN/07 | EU910866 | Phylogenetic analysis | GenBank |
| 2007 | Inner Mongolia | 0717F/NM/CHN/07 | EU910867 | Phylogenetic analysis | GenBank |
| 2007 | Inner Mongolia | 0718F/NM/CHN/07 | EU910868 | Phylogenetic analysis | GenBank |
| 2007 | Inner Mongolia | 0723F/NM/CHN/07 | EU910869 | Phylogenetic & Evolutionary analysis | GenBank |
| 2007 | Shandong | 518-01F/SD/CHN/07 | EU753363 | Phylogenetic analysis | GenBank |
| 2007 | Shandong | 518-02F/SD/CHN/07 | EU753364 | Phylogenetic analysis | GenBank |
| 2007 | Shandong | 518-03F/SD/CHN/07 | EU753365 | Phylogenetic analysis | GenBank |
| 2007 | Shandong | 519-02F/SD/CHN/07 | EU753366 | Phylogenetic analysis | GenBank |
| 2007 | Shandong | 521-03T/SD/CHN/07 | EU753367 | Phylogenetic analysis | GenBank |
| 2007 | Shandong | 521-04F/SD/CHN/07 | EU753368 | Phylogenetic analysis | GenBank |
| 2007 | Shandong | 521-04T/SD/CHN/07 | EU753369 | Phylogenetic analysis | GenBank |
| 2007 | Shandong | 521-05T/SD/CHN/07 | EU753370 | Phylogenetic analysis | GenBank |
| 2007 | Shandong | 521-08F/SD/CHN/07 | EU753371 | Phylogenetic analysis | GenBank |
| 2007 | Shandong | 521-09F/SD/CHN/07 | EU753372 | Phylogenetic analysis | GenBank |
| 2007 | Shandong | 521-14F/SD/CHN/07 | EU753373 | Phylogenetic analysis | GenBank |
| 2007 | Shandong | 521-17F/SD/CHN/07 | EU753374 | Phylogenetic analysis | GenBank |
| 2007 | Shandong | 521-18S/SD/CHN/07 | EU753375 | Phylogenetic analysis | GenBank |
| 2007 | Shandong | 521-19T/SD/CHN/07 | EU753376 | Phylogenetic analysis | GenBank |
| 2007 | Shandong | 521-20F/SD/CHN/07 | EU753377 | Phylogenetic analysis | GenBank |
| 2007 | Shandong | 521-23F/SD/CHN/07 | EU753378 | Phylogenetic analysis | GenBank |
| 2007 | Shandong | 521-25F/SD/CHN/07 | EU753379 | Phylogenetic analysis | GenBank |
| 2007 | Shandong | 521-27F/SD/CHN/07 | EU753380 | Phylogenetic analysis | GenBank |
| 2007 | Shandong | 522-01T/SD/CHN/07 | EU753381 | Phylogenetic analysis | GenBank |
| 2007 | Shandong | 522-02F/SD/CHN/07 | EU753382 | Phylogenetic analysis | GenBank |
| 2007 | Shandong | 522-03F/SD/CHN/07 | EU753383 | Phylogenetic analysis | GenBank |
| 2007 | Shandong | 522-04T/SD/CHN/07 | EU753384 | Phylogenetic analysis | GenBank |
| 2007 | Shandong | 522-07T/SD/CHN/07 | EU753385 | Phylogenetic analysis | GenBank |
| 2007 | Shandong | 522-08T/SD/CHN/07 | EU753386 | Phylogenetic analysis | GenBank |
| 2007 | Shandong | 522-16F/SD/CHN/07 | EU753387 | Phylogenetic analysis | GenBank |
| 2007 | Shandong | 522-17F/SD/CHN/07 | EU753388 | Phylogenetic analysis | GenBank |
| 2007 | Shandong | 522-17T/SD/CHN/07 | EU753389 | Phylogenetic analysis | GenBank |
| 2007 | Shandong | 522-18T/SD/CHN/07 | EU753390 | Phylogenetic analysis | GenBank |
| 2007 | Shandong | 522-19F/SD/CHN/07 | EU753391 | Phylogenetic analysis | GenBank |
| 2007 | Shandong | 522-21F/SD/CHN/07 | EU753392 | Phylogenetic analysis | GenBank |
| 2007 | Shandong | 522-23F/SD/CHN/07 | EU753393 | Phylogenetic analysis | GenBank |
| 2007 | Shandong | 522-24F/SD/CHN/07 | EU753394 | Phylogenetic analysis | GenBank |
| 2007 | Shandong | 523-03F/SD/CHN/07 | EU753395 | Phylogenetic analysis | GenBank |
| 2007 | Shandong | 523-04F/SD/CHN/07 | EU753396 | Phylogenetic analysis | GenBank |
| 2007 | Shandong | 523-05T/SD/CHN/07 | EU753397 | Phylogenetic analysis | GenBank |
| 2007 | Shandong | 523-07T/SD/CHN/07 | EU753398 | Phylogenetic & Evolutionary analysis | GenBank |
| 2007 | Shandong | 523-10F/SD/CHN/07 | EU753399 | Phylogenetic analysis | GenBank |
| 2007 | Shandong | 523-10T/SD/CHN/07 | EU753400 | Phylogenetic analysis | GenBank |
| 2007 | Shandong | 523-11F/SD/CHN/07 | EU753401 | Phylogenetic analysis | GenBank |
| 2007 | Shandong | 523-11T/SD/CHN/07 | EU753402 | Phylogenetic & Evolutionary analysis | GenBank |
| 2007 | Shandong | 523-13T/SD/CHN/07 | EU753403 | Phylogenetic & Evolutionary analysis | GenBank |
| 2007 | Shandong | 523-14T/SD/CHN/07 | EU753404 | Phylogenetic analysis | GenBank |
| 2007 | Shandong | 523-15F/SD/CHN/07 | EU753405 | Phylogenetic analysis | GenBank |
| 2007 | Shandong | 523-15T/SD/CHN/07 | EU753406 | Phylogenetic analysis | GenBank |
| 2007 | Shandong | TC03F/SD/CHN/07 | EU753407 | Phylogenetic analysis | GenBank |
| 2007 | Shandong | TC06T/SD/CHN/07 | EU753408 | Phylogenetic analysis | GenBank |
| 2007 | Shandong | TC08F/SD/CHN/07 | EU753409 | Phylogenetic analysis | GenBank |
| 2007 | Shandong | TC09F/SD/CHN/07 | EU753410 | Phylogenetic analysis | GenBank |
| 2007 | Shandong | TC14F/SD/CHN/07 | EU753411 | Phylogenetic analysis | GenBank |
| 2007 | Shandong | TC14T/SD/CHN/07 | EU753412 | Phylogenetic analysis | GenBank |
| 2007 | Shandong | TC16T/SD/CHN/07 | EU753413 | Phylogenetic analysis | GenBank |
| 2007 | Shandong | TC20F/SD/CHN/07 | EU753414 | Phylogenetic analysis | GenBank |
| 2007 | Shandong | TC20T/SD/CHN/07 | EU753415 | Phylogenetic analysis | GenBank |
| 2007 | Shandong | TC22F/SD/CHN/07 | EU753416 | Phylogenetic analysis | GenBank |
| 2007 | Shandong | TC23F/SD/CHN/07 | EU753417 | Phylogenetic analysis | GenBank |
| 2007 | Shandong | TC24F/SD/CHN/07 | EU753418 | Phylogenetic analysis | GenBank |
| 2008 | Anhui | 542-Anhui-08/2008 | FJ765416 | Phylogenetic analysis | GenBank |
| 2008 | Anhui | 549-Anhui-08/2008 | FJ765417 | Phylogenetic analysis | GenBank |
| 2008 | Anhui | 552-Anhui-08/2008 | FJ765418 | Phylogenetic analysis | GenBank |
| 2008 | Anhui | 559-Anhui-08/2008 | FJ765419 | Phylogenetic analysis | GenBank |
| 2008 | Anhui | 562-Anhui-08/2008 | FJ765420 | Phylogenetic analysis | GenBank |
| 2008 | Anhui | 566-Anhui-08/2008 | FJ765421 | Phylogenetic & Evolutionary analysis | GenBank |
| 2008 | Anhui | EV71/Fuyang.Anhui.P.R.C/17.08/1 | EU703812 | Phylogenetic analysis | GenBank |
| 2008 | Anhui | EV71/Fuyang.Anhui.P.R.C/17.08/2 | EU703813 | Phylogenetic analysis | GenBank |
| 2008 | Anhui | EV71/Fuyang.Anhui.P.R.C/17.08/3 | EU703814 | Phylogenetic & Evolutionary analysis | GenBank |
| 2008 | Anhui | Fuyang-0805 | FJ439769 | Phylogenetic analysis | GenBank |
| 2008 | Anhui | Fuyang-0805a | GU350629 | Phylogenetic analysis | GenBank |
| 2008 | Anhui | Fuyang22 | EU913466 | Phylogenetic analysis | GenBank |
| 2008 | Anhui | Fuyang26 | EU913468 | Phylogenetic analysis | GenBank |
| 2008 | Anhui | Fuyang31 | EU913470 | Phylogenetic analysis | GenBank |
| 2008 | Anhui | Fuyang44 | EU913469 | Phylogenetic analysis | GenBank |
| 2008 | Anhui | Fuyang49 | EU913471 | Phylogenetic analysis | GenBank |
| 2008 | Anhui | Fuyang5 | EU913467 | Phylogenetic analysis | GenBank |
| 2008 | Anhui | FY08-16/AH/CHN/2008 | HM212458 | Phylogenetic analysis | this study |
| 2008 | Anhui | FY08-4/AH/CHN/2008 | HM212456 | Phylogenetic analysis | this study |
| 2008 | Anhui | FY08-7/AH/CHN/2008 | HM212455 | Phylogenetic & Evolutionary analysis | this study |
| 2008 | Anhui | FY08-9/AH/CHN/2008 | HM212457 | Phylogenetic analysis | this study |
| 2008 | Anhui | FY23 | EU812515 | Phylogenetic & Evolutionary analysis | GenBank |
| 2008 | Beijing | BJ08 | FJ828519 | Phylogenetic & Evolutionary analysis | GenBank |
| 2008 | Beijing | BJ08-Z004-3 | FJ606447 | Phylogenetic analysis | GenBank |
| 2008 | Beijing | BJ08-Z011-4 | FJ606448 | Phylogenetic analysis | GenBank |
| 2008 | Beijing | BJ08-Z020-1 | FJ606449 | Phylogenetic analysis | GenBank |
| 2008 | Beijing | BJ08-Z025-5 | FJ606450 | Phylogenetic & Evolutionary analysis | GenBank |
| 2008 | Beijing | BJCDC01-08/2008 | FJ765422 | Phylogenetic & Evolutionary analysis | GenBank |
| 2008 | Beijing | BJCDC03-08/2008 | FJ765423 | Phylogenetic & Evolutionary analysis | GenBank |
| 2008 | Beijing | CY11/BJ/CHN/2008 | FJ469153 | Phylogenetic analysis | GenBank |
| 2008 | Beijing | CY15/BJ/CHN/2008 | FJ469154 | Phylogenetic analysis | GenBank |
| 2008 | Beijing | CY17/BJ/CHN/2008 | FJ469155 | Phylogenetic analysis | GenBank |
| 2008 | Beijing | CY20/BJ/CHN/2008 | FJ469156 | Phylogenetic analysis | GenBank |
| 2008 | Beijing | CY21/BJ/CHN/2008 | FJ469157 | Phylogenetic analysis | GenBank |
| 2008 | Beijing | CY28/BJ/CHN/2008 | FJ469158 | Phylogenetic analysis | GenBank |
| 2008 | Beijing | Cy29/BJ/CHN/2008 | FJ469159 | Phylogenetic analysis | GenBank |
| 2008 | Beijing | CY43/BJ/CHN/2008 | FJ469160 | Phylogenetic analysis | GenBank |
| 2008 | Beijing | CY44/BJ/CHN/2008 | FJ469161 | Phylogenetic analysis | GenBank |
| 2008 | Beijing | CY6/BJ/CHN/2008 | FJ469152 | Phylogenetic & Evolutionary analysis | GenBank |
| 2008 | Gansu | EV71/Lanzhou01 | GQ855285 | Phylogenetic analysis | GenBank |
| 2008 | Gansu | EV71/Lanzhou02 | GQ855286 | Phylogenetic analysis | GenBank |
| 2008 | Gansu | EV71/Lanzhou03 | GQ855287 | Phylogenetic & Evolutionary analysis | GenBank |
| 2008 | Gansu | EV71/Lanzhou04 | GQ855288 | Phylogenetic analysis | GenBank |
| 2008 | Gansu | EV71/Lanzhou05 | GQ855289 | Phylogenetic & Evolutionary analysis | GenBank |
| 2008 | Gansu | EV71/Lanzhou06 | GQ855290 | Phylogenetic analysis | GenBank |
| 2008 | Gansu | EV71/Lanzhou07 | GQ855291 | Phylogenetic analysis | GenBank |
| 2008 | Gansu | EV71/Lanzhou08 | GQ855292 | Phylogenetic analysis | GenBank |
| 2008 | Gansu | EV71/Lanzhou09 | GQ855293 | Phylogenetic analysis | GenBank |
| 2008 | Gansu | EV71/Lanzhou10 | GQ855294 | Phylogenetic analysis | GenBank |
| 2008 | Guangdong | 1/GZ/CHN/2008 | GU190169 | Phylogenetic analysis | GenBank |
| 2008 | Guangdong | 1/SHENZHEN/08/China/HFMD/2008 | FJ607334 | Phylogenetic analysis | GenBank |
| 2008 | Guangdong | 121/SHENZHEN/08/China/HFMD Fatal/2008 | FJ607337 | Phylogenetic & Evolutionary analysis | GenBank |
| 2008 | Guangdong | 122/GZ/CHN/2008 | GU190170 | Phylogenetic analysis | GenBank |
| 2008 | Guangdong | 129/GZ/CHN/2008 | GU190171 | Phylogenetic analysis | GenBank |
| 2008 | Guangdong | 130/GZ/CHN/2008 | GU190172 | Phylogenetic analysis | GenBank |
| 2008 | Guangdong | 133/GZ/CHN/2008 | GU190173 | Phylogenetic analysis | GenBank |
| 2008 | Guangdong | 136/GZ/CHN/2008 | GU190174 | Phylogenetic analysis | GenBank |
| 2008 | Guangdong | 142/GZ/CHN/2008 | GU190175 | Phylogenetic analysis | GenBank |
| 2008 | Guangdong | 145/GZ/CHN/2008 | GU190176 | Phylogenetic analysis | GenBank |
| 2008 | Guangdong | 152/GZ/CHN/2008 | GU190177 | Phylogenetic analysis | GenBank |
| 2008 | Guangdong | 168/GZ/CHN/2008 | GU190178 | Phylogenetic analysis | GenBank |
| 2008 | Guangdong | 28/SHENZHEN/08/China/HFMD/2008 | FJ607336 | Phylogenetic analysis | GenBank |
| 2008 | Guangdong | 4/SHENZHEN/08/China/HFMD/2008 | FJ607335 | Phylogenetic analysis | GenBank |
| 2008 | Guangdong | 605/SHENZHEN/08/China/HFMD Severe/2008 | FJ607338 | Phylogenetic & Evolutionary analysis | GenBank |
| 2008 | Guangdong | EV71/GDFS/3/2008 | FJ194964 | Phylogenetic & Evolutionary analysis | GenBank |
| 2008 | Guangdong | EV71/GDSG/17/2008 | FJ194965 | Phylogenetic & Evolutionary analysis | GenBank |
| 2008 | Guangdong | GZ-08-01 | FJ360544 | Phylogenetic analysis | GenBank |
| 2008 | Guangdong | GZ-08-02 | FJ360545 | Phylogenetic analysis | GenBank |
| 2008 | Guangdong | GZ-08-03 | FJ360546 | Phylogenetic & Evolutionary analysis | GenBank |
| 2008 | Guangdong | SZ-A-08/2008 | FJ765428 | Phylogenetic analysis | GenBank |
| 2008 | Guangdong | SZ-C-08/2008 | FJ765429 | Phylogenetic analysis | GenBank |
| 2008 | Guangdong | SZ-H-08/2008 | FJ765430 | Phylogenetic & Evolutionary analysis | GenBank |
| 2008 | Guangdong | Xinhui-7 | EU999177 | Phylogenetic analysis | GenBank |
| 2008 | Guangdong | Xinhui-8 | EU999178 | Phylogenetic analysis | GenBank |
| 2008 | Guangdong | Xinhui-9 | EU999179 | Phylogenetic & Evolutionary analysis | GenBank |
| 2008 | Guangdong | Zhuhai-152 | EU999176 | Phylogenetic analysis | GenBank |
| 2008 | Guangdong | Zhuhai-164 | EU999175 | Phylogenetic analysis | GenBank |
| 2008 | Guangdong | Zhuhai-171 | EU999174 | Phylogenetic analysis | GenBank |
| 2008 | Guangdong | Zhuhai-213 | EU999173 | Phylogenetic analysis | GenBank |
| 2008 | Guangdong | Zhuhai-JC455 | EU999171 | Phylogenetic analysis | GenBank |
| 2008 | Guangdong | Zhuhai-JC467 | EU999170 | Phylogenetic & Evolutionary analysis | GenBank |
| 2008 | Guangdong | Zhuhai-JC498 | EU999172 | Phylogenetic analysis | GenBank |
| 2008 | Guangxi | GX/LZ 08-04/08/CHN | GQ892830 | Phylogenetic & Evolutionary analysis | GenBank |
| 2008 | Hebei | 11/HeB/CHN/2008 | HM212445 | Phylogenetic & Evolutionary analysis | this study |
| 2008 | Hebei | 20/HeB/CHN/2008 | HM212446 | Phylogenetic analysis | this study |
| 2008 | Hebei | 24/HeB/CHN/2008 | HM212447 | Phylogenetic analysis | this study |
| 2008 | Hebei | 40/HeB/CHN/2008 | HM212448 | Phylogenetic & Evolutionary analysis | this study |
| 2008 | Hebei | 48/HeB/CHN/2008 | HM212449 | Phylogenetic analysis | this study |
| 2008 | Hebei | 50/HeB/CHN/2008 | HM212450 | Phylogenetic analysis | this study |
| 2008 | Hebei | 9/HeB/CHN/2008 | HM212444 | Phylogenetic analysis | this study |
| 2008 | Henan | 08-8/HeN/CHN/2008 | HM212451 | Phylogenetic & Evolutionary analysis | this study |
| 2008 | Henan | Henan10-08-China | GU366191 | Phylogenetic analysis | GenBank |
| 2008 | Henan | HN08-HLF2 | GQ121133 | Phylogenetic analysis | GenBank |
| 2008 | Henan | HN08-HLF3 | GQ121134 | Phylogenetic analysis | GenBank |
| 2008 | Hubei | WH-2-08/2008 | FJ765431 | Phylogenetic & Evolutionary analysis | GenBank |
| 2008 | Hubei | WH-3-08/2008 | FJ765432 | Phylogenetic analysis | GenBank |
| 2008 | Hubei | WH-4-08/2008 | FJ765433 | Phylogenetic analysis | GenBank |
| 2008 | Hubei | WH-8-08/2008 | FJ765434 | Phylogenetic analysis | GenBank |
| 2008 | Hubei | WH-9-08/2008 | FJ765435 | Phylogenetic analysis | GenBank |
| 2008 | Jiangsu | EV71/Jiangsu.P.R.C/07.08/10 | FJ600325 | Phylogenetic & Evolutionary analysis | GenBank |
| 2008 | Jiangsu | Nanjing08-1 | GU176408 | Phylogenetic & Evolutionary analysis | GenBank |
| 2008 | Jilin | 08-2/JL/CHN/2008 | HM212452 | Phylogenetic & Evolutionary analysis | this study |
| 2008 | Jilin | 08-7/JL/CHN/2008 | HM212453 | Phylogenetic analysis | this study |
| 2008 | Jilin | 08-8/JL/CHN/2008 | HM212454 | Phylogenetic analysis | this study |
| 2008 | Jilin | CC01-08/2008 | FJ765424 | Phylogenetic & Evolutionary analysis | GenBank |
| 2008 | Ningxia | 082/NX/CHN/2008 | HM212460 | Phylogenetic analysis | this study |
| 2008 | Ningxia | 117/NX/CHN/2008 | HM212461 | Phylogenetic analysis | this study |
| 2008 | Ningxia | 174/NX/CHN/2008 | HM212462 | Phylogenetic analysis | this study |
| 2008 | Ningxia | 201/NX/CHN/2008 | HM212463 | Phylogenetic analysis | this study |
| 2008 | Ningxia | 207/NX/CHN/2008 | HM212464 | Phylogenetic analysis | this study |
| 2008 | Shandong | H027F/SD/CHN/2008 | GQ253391 | Phylogenetic analysis | GenBank |
| 2008 | Shandong | H1218F/SD/CHN/2008 | GQ253398 | Phylogenetic analysis | GenBank |
| 2008 | Shandong | H1261F/SD/CHN/2008 | GQ253399 | Phylogenetic & Evolutionary analysis | GenBank |
| 2008 | Shandong | H150F/SD/CHN/2008 | GQ253392 | Phylogenetic analysis | GenBank |
| 2008 | Shandong | H362F/SD/CHN/2008 | GQ253393 | Phylogenetic analysis | GenBank |
| 2008 | Shandong | H419F/SD/CHN/2008 | GQ253394 | Phylogenetic analysis | GenBank |
| 2008 | Shandong | H533F/SD/CHN/2008 | GQ253395 | Phylogenetic analysis | GenBank |
| 2008 | Shandong | H553F/SD/CHN/2008 | GQ253396 | Phylogenetic analysis | GenBank |
| 2008 | Shandong | H903F/SD/CHN/2008 | GQ253397 | Phylogenetic analysis | GenBank |
| 2008 | Yunnan | 08-5/YN/CHN/2008 | HM212459 | Phylogenetic & Evolutionary analysis | this study |
| 2008 | Yunnan | kunming24-08/2008 | FJ765425 | Phylogenetic analysis | GenBank |
| 2008 | Yunnan | kunming29-08/2008 | FJ765426 | Phylogenetic analysis | GenBank |
| 2008 | Yunnan | kunming41-08/2008 | FJ765427 | Phylogenetic & Evolutionary analysis | GenBank |
| 2008 | Zhejiang | DTID/ZJU-62 | FJ158600 | Phylogenetic analysis | GenBank |
| 2008 | Zhejiang | DTID/ZJU-74 | FJ158601 | Phylogenetic & Evolutionary analysis | GenBank |
| 2008 | Zhejiang | EV71/Zhejiang08 | EU864507 | Phylogenetic & Evolutionary analysis | GenBank |
| 2009 | Anhui | Anhui1-09-China | GQ994988 | Phylogenetic & Evolutionary analysis | GenBank |
| 2009 | Beijing | Beijing0907 | GQ406340 | Phylogenetic analysis | GenBank |
| 2009 | Beijing | Beijing0907 | GQ406338 | Phylogenetic analysis | GenBank |
| 2009 | Chongqing | Chongqing1-09-China | GQ994989 | Phylogenetic & Evolutionary analysis | GenBank |
| 2009 | Chongqing | Chongqing2-09-China | GQ994990 | Phylogenetic analysis | GenBank |
| 2009 | Chongqing | Chongqing3-09-China | GQ994991 | Phylogenetic analysis | GenBank |
| 2009 | Gansu | EV71/Lanzhou01 | GU396280 | Phylogenetic analysis | GenBank |
| 2009 | Guangdong | 373/GZ/CHN/2009 | GU190179 | Phylogenetic & Evolutionary analysis | GenBank |
| 2009 | Guangdong | ZH-ETC110/GD/CHN/09 | GQ487684 | Phylogenetic analysis | GenBank |
| 2009 | Guangdong | ZH-ETC153/GD/CHN/09 | GQ487672 | Phylogenetic analysis | GenBank |
| 2009 | Guangdong | ZH-ETC180/GD/CHN/09 | GQ487683 | Phylogenetic analysis | GenBank |
| 2009 | Guangdong | ZH-ETC222/GD/CHN/09 | GQ487671 | Phylogenetic analysis | GenBank |
| 2009 | Guangdong | ZH-ETC275/GD/CHN/09 | GQ487681 | Phylogenetic & Evolutionary analysis | GenBank |
| 2009 | Guangdong | ZH-ETC278/GD/CHN/09 | GQ487669 | Phylogenetic analysis | GenBank |
| 2009 | Guangdong | ZH-ETC287/GD/CHN/09 | GQ487680 | Phylogenetic analysis | GenBank |
| 2009 | Guangdong | ZH-ETC321/GD/CHN/09 | GQ487679 | Phylogenetic & Evolutionary analysis | GenBank |
| 2009 | Guangdong | ZH-ETC335/GD/CHN/09 | GQ487667 | Phylogenetic analysis | GenBank |
| 2009 | Guangdong | ZH-ETC346/GD/CHN/09 | GQ487687 | Phylogenetic analysis | GenBank |
| 2009 | Guangdong | ZH-ETC351/GD/CHN/09 | GQ487678 | Phylogenetic analysis | GenBank |
| 2009 | Guangdong | ZH-ETC352/GD/CHN/09 | GQ487674 | Phylogenetic & Evolutionary analysis | GenBank |
| 2009 | Guangdong | ZH-ETC367/GD/CHN/09 | GQ487685 | Phylogenetic analysis | GenBank |
| 2009 | Guangdong | ZH-ETC368/GD/CHN/09 | GQ487673 | Phylogenetic analysis | GenBank |
| 2009 | Guangdong | ZH-ETC377/GD/CHN/09 | GQ487666 | Phylogenetic analysis | GenBank |
| 2009 | Guangdong | ZH-ETC385/GD/CHN/09 | GQ487686 | Phylogenetic analysis | GenBank |
| 2009 | Guangdong | ZH-JC209/GD/CHN/09 | GQ487682 | Phylogenetic analysis | GenBank |
| 2009 | Guangdong | ZH-JC215/GD/CHN/09 | GQ487670 | Phylogenetic analysis | GenBank |
| 2009 | Guangdong | ZH-JC220/GD/CHN/09 | GQ487668 | Phylogenetic analysis | GenBank |
| 2009 | Guangdong | ZH-JC334/GD/CHN/09 | GQ487688 | Phylogenetic analysis | GenBank |
| 2009 | Guangdong | ZH-JC344/GD/CHN/09 | GQ487675 | Phylogenetic analysis | GenBank |
| 2009 | Guangdong | ZH-JC414/GD/CHN/09 | GQ487677 | Phylogenetic analysis | GenBank |
| 2009 | Guangdong | ZH-JC426/GD/CHN/09 | GQ487689 | Phylogenetic analysis | GenBank |
| 2009 | Guangdong | ZH-JC431/GD/CHN/09 | GQ487676 | Phylogenetic analysis | GenBank |
| 2009 | Henan | 09-1/HeN/CHN/2009 | HM212465 | Phylogenetic & Evolutionary analysis | this study |
| 2009 | Henan | 09-17/HeN/CHN/2009 | HM212466 | Phylogenetic analysis | this study |
| 2009 | Henan | Henan1-09-China | GU196833 | Phylogenetic & Evolutionary analysis | GenBank |
| 2009 | Henan | Henan2-09-China | GQ994992 | Phylogenetic & Evolutionary analysis | GenBank |
| 2009 | Hubei | 09-20/HuB/CHN/2009 | HM212436 | Phylogenetic analysis | this study |
| 2009 | Hubei | 09-29H/HuB/CHN/2009 | HM212437 | Phylogenetic & Evolutionary analysis | this study |
| 2009 | Hubei | 09-32H/HuB/CHN/2009 | HM212438 | Phylogenetic analysis | this study |
| 2009 | Hubei | 09-33T/HuB/CHN/2009 | HM212439 | Phylogenetic & Evolutionary analysis | this study |
| 2009 | Hubei | 09-40H/HuB/CHN/2009 | HM212440 | Phylogenetic analysis | this study |
| 2009 | Jiangsu | Changzhou/JS10/CHN09 | GU353081 | Phylogenetic analysis | GenBank |
| 2009 | Jiangsu | Lianyungang/JS12/CHN09 | GU353082 | Phylogenetic analysis | GenBank |
| 2009 | Jiangsu | Lianyungang/JS13/CHN09 | GU353083 | Phylogenetic analysis | GenBank |
| 2009 | Jiangsu | Lianyungang/JS14/CHN09 | GU353084 | Phylogenetic analysis | GenBank |
| 2009 | Jiangsu | Lianyungang/JS24/CHN09 | GU353090 | Phylogenetic analysis | GenBank |
| 2009 | Jiangsu | Nanjing/JS05/CHN09 | GU353079 | Phylogenetic & Evolutionary analysis | GenBank |
| 2009 | Jiangsu | Nanjing/JS06/CHN09 | GU353080 | Phylogenetic analysis | GenBank |
| 2009 | Jiangsu | Nantong/JS15/CHN09 | GU353085 | Phylogenetic analysis | GenBank |
| 2009 | Jiangsu | Nantong/JS16/CHN09 | GU353086 | Phylogenetic analysis | GenBank |
| 2009 | Jiangsu | Nantong/JS23/CHN09 | GU353089 | Phylogenetic analysis | GenBank |
| 2009 | Jiangsu | Nantong/JS29/CHN09 | GU353091 | Phylogenetic analysis | GenBank |
| 2009 | Jiangsu | Suqian/JS22/CHN09 | GU353088 | Phylogenetic analysis | GenBank |
| 2009 | Jiangsu | Suqian/JS34/CHN09 | GU353093 | Phylogenetic analysis | GenBank |
| 2009 | Jiangsu | Suzhou/JS30/CHN09 | GU353092 | Phylogenetic analysis | GenBank |
| 2009 | Jiangsu | Taizhou/JS18/CHN09 | GU353087 | Phylogenetic analysis | GenBank |
| 2009 | Jiangsu | Yangzhou/JS35/CHN09 | GU353094 | Phylogenetic analysis | GenBank |
| 2009 | Jiangsu | Yangzhou/JS36/CHN09 | GU353095 | Phylogenetic analysis | GenBank |
| 2009 | Jiangsu | Zhenjiang/JS41/CHN09 | GU353096 | Phylogenetic analysis | GenBank |
| 2009 | Jiangsu | Zhenjiang/JS42/CHN09 | GU353097 | Phylogenetic analysis | GenBank |
| 2009 | Jiangsu | Zhenjiang/JS43/CHN09 | GU353098 | Phylogenetic analysis | GenBank |
| 2009 | Jiangsu | Zhenjiang/JS44/CHN09 | GU353099 | Phylogenetic analysis | GenBank |
| 2009 | Jiangsu | Zhenjiang/JS45/CHN09 | GU353100 | Phylogenetic analysis | GenBank |
| 2009 | Jiangsu | Zhenjiang/JS46/CHN09 | GU353101 | Phylogenetic analysis | GenBank |
| 2009 | Jiangsu | Zhenjiang/JS51/CHN09 | GU353102 | Phylogenetic analysis | GenBank |
| 2009 | Jiangsu | Zhenjiang/JS56/CHN09 | GU353103 | Phylogenetic analysis | GenBank |
| 2009 | Jiangsu | Zhenjiang/JS57/CHN09 | GU353104 | Phylogenetic analysis | GenBank |
| 2009 | Jiangsu | Zhenjiang/JS59/CHN09 | GU353105 | Phylogenetic analysis | GenBank |
| 2009 | Jiangsu | Zhenjiang/JS60/CHN09 | GU353106 | Phylogenetic & Evolutionary analysis | GenBank |
| 2009 | Shandong | HZ0011F/SD/CHN/2009 | GQ253403 | Phylogenetic analysis | GenBank |
| 2009 | Shandong | HZ0048F/SD/CHN/2009 | GQ253404 | Phylogenetic analysis | GenBank |
| 2009 | Shandong | HZ0137F/SD/CHN/2009 | GQ253400 | Phylogenetic analysis | GenBank |
| 2009 | Shandong | HZ121F/SD/CHN/2009 | GQ253405 | Phylogenetic analysis | GenBank |
| 2009 | Shandong | HZ150F/SD/CHN/2009 | GQ253401 | Phylogenetic analysis | GenBank |
| 2009 | Shandong | HZ152F/SD/CHN/2009 | GQ253402 | Phylogenetic analysis | GenBank |
| 2009 | Shandong | JN92F/SD/CHN/2009 | GQ253406 | Phylogenetic analysis | GenBank |
| 2009 | Shandong | JN93F/SD/CHN/2009 | GQ253407 | Phylogenetic analysis | GenBank |
| 2009 | Shandong | JN94F/SD/CHN/2009 | GQ253408 | Phylogenetic analysis | GenBank |
| 2009 | Shandong | LC0013H/SD/CHN/2009 | GQ253409 | Phylogenetic analysis | GenBank |
| 2009 | Shandong | LC0028F/SD/CHN/2009 | GQ253410 | Phylogenetic analysis | GenBank |
| 2009 | Shandong | LW0004F/SD/CHN/2009 | GQ253411 | Phylogenetic analysis | GenBank |
| 2009 | Shandong | LW0009F/SD/CHN/2009 | GQ253412 | Phylogenetic analysis | GenBank |
| 2009 | Shandong | LW0011F/SD/CHN/2009 | GQ253413 | Phylogenetic analysis | GenBank |
| 2009 | Shandong | LY0004F/SD/CHN/2009 | GQ253414 | Phylogenetic analysis | GenBank |
| 2009 | Shandong | LY0009F/SD/CHN/2009 | GQ253415 | Phylogenetic analysis | GenBank |
| 2009 | Shandong | RZ0008F/SD/CHN/2009 | GQ253416 | Phylogenetic analysis | GenBank |
| 2009 | Shandong | RZ0010F/SD/CHN/2009 | GQ253417 | Phylogenetic analysis | GenBank |
| 2009 | Shandong | ZB0001F/SD/CHN/2009 | GQ253418 | Phylogenetic analysis | GenBank |
| 2009 | Shandong | ZB0002F/SD/CHN/2009 | GQ253419 | Phylogenetic & Evolutionary analysis | GenBank |
| 2009 | Shanghai | Shanghai 036-2009 | FJ713137 | Phylogenetic & Evolutionary analysis | GenBank |
