# Supplementary material for: The Persistent Circulation of Enterovirus 71 in People's Republic of China: Causing Emerging Nationwide Epidemics Since 2008
Source: PLoS One. 2011 Sep 28;6(9):e25662. doi: 10.1371/journal.pone.0025662 (PMC3181342; doi:10.1371/journal.pone.0025662)
Supplement: Table S2 — Reference strains used for phylogenetic analysis (CA16 was used as out-group control). (DOC) [file pone.0025662.s002.doc]

| Strains | Isolated year | isolated region | | | Genotype/Subgenotype | GenBank No. |
| --- | --- | --- | --- | --- | --- | --- |
| 8495-VA-88 | 1988 | Virginia | | | C1 | AF135953 |
| 0925-OR-91 | 1991 | Oregon | | | C1 | AF009547 |
| 2623-AUS-86 | 1986 | Australia | | | C1 | AF135945 |
| 0915-MA-87 | 1987 | Massachusetts | | | C1 | AF009549 |
| 2253-NY-94 | 1994 | New York | | | C1 | AF009544 |
| 0443-TX-90 | 1990 | Texas | | | C1 | AF135933 |
| 0389-MAA-00 | 2000 | Malaysia | | | C1 | AY207626 |
| 4575/SIN/98 | 1998 | Singapore | | | C1 | AF376120 |
| 2644-AUS-95 | 1995 | Australia | | | C2 | AF135949 |
| 03907-MAA-97 | 1997 | Malaysia | | | C2 | AY207611 |
| 2286-TX-97 | 1997 | Texas | | | C2 | AF135941 |
| 2912-TAI-98 | 1998 | China Taiwan | | | C2 | AF286522 |
| 2885-TAI-98 | 1998 | China Taiwan | | | C2 | AF286512 |
| 2915-TAI-98 | 1998 | China Taiwan | | | C2 | AF286525 |
| KOR-EV71-05 | 2000 | Korea | | | C3 | AY125969 |
| KOR-EV71-06 | 2000 | Korea | | | C3 | AY125970 |
| KOR-EV71-01 | 2000 | Korea | | | C3 | AY125966 |
| KOR-EV71-02 | 2000 | Korea | | | C3 | AY125967 |
| KOR-EV71-07 | 2000 | Korea | | | C3 | AY125971 |
| KOR-EV71-13 | 2000 | Korea | | | C3 | AY125976 |
| 3254-TAI-98 | 1998 | China Taiwan | | | C4 | AF286531 |
| SI01/TH(NMA)/06 | 2006 | | Thailand | C4 | | EF203407 |
| SHZH98 | 1998 | China | | | C4 | AF302996 |
| 933V/VNM/05 | 2005 | Vietnam | | | C5 | AM490161 |
| 1301V/VNM/05 | 2005 | Vietnam | | | C5 | AM490149 |
| 1192S/VNM/05 | 2005 | Vietnam | | | C5 | AM490147 |
| BrCr-CA-70 | 1970 | California | | | A | U22521 |
| 7234-AK-87 | 1987 | Alaska | | | B | AF009522 |
| 1413-CA-87 | 1987 | California | | | B | AF009527 |
| MS/7423/87 | 1987 | Mississippi | | | B | U22522 |
| 2605-AUS-74 | 1974 | Australia | | | B | AF135884 |
| 2604-AUS-74 | 1974 | Australia | | | B | AF135883 |
| 2608-AUS-74 | 1974 | Australia | | | B | AF135885 |
| CA16-G10 | NA | South Africa | | | Out-group control | U05876 |
