# Supplementary material for: The Persistent Circulation of Enterovirus 71 in People's Republic of China: Causing Emerging Nationwide Epidemics Since 2008
Source: PLoS One. 2011 Sep 28;6(9):e25662. doi: 10.1371/journal.pone.0025662 (PMC3181342; doi:10.1371/journal.pone.0025662)
Supplement: Table S3 — The locality and time distribution of the EV71 VP1 sequences involved in this study. (DOC) [file pone.0025662.s003.doc]

| Province | Numbers of sequences | | | | | | | | | | | |
| --- | --- | --- | --- | --- | --- | --- | --- | --- | --- | --- | --- | --- |
|  | 1987~1999 | 2000 | 2001 | 2002 | 2003 | 2004 | 2005 | 2006 | 2007 | 2008 | 2009 | Total |
| Hubei | 1 |  |  |  |  |  |  |  |  | 5 | 5 | 11 |
| Heilongjiang | 1 |  |  |  |  |  |  |  |  |  |  | 1 |
| Guangdong | 1 |  | 2 | 2 | 4 | 11 |  |  |  | 33 | 25 | 78 |
| Shanghai |  | 4 |  | 2 |  |  |  |  |  |  | 1 | 7 |
| Chongqing |  |  |  |  | 1 |  |  |  |  |  | 3 | 4 |
| Zhejiang |  |  |  |  | 6 |  |  |  |  | 3 |  | 9 |
| Shandong |  |  |  |  | 3 |  | 2 | 2 | 56 | 9 | 20 | 92 |
| Inner Mongolia |  |  |  |  |  |  |  |  | 9 |  |  | 9 |
| Beijing |  |  |  |  |  |  |  |  | 2 | 17 | 2 | 21 |
| Anhui |  |  |  |  |  |  |  | 1 |  | 22 | 1 | 28 |
| Gansu |  |  |  |  |  |  |  |  |  | 10 | 1 | 11 |
| Guangxi |  |  |  |  |  |  |  |  |  | 1 |  | 1 |
| Hebei |  |  |  |  |  |  |  |  |  | 7 |  | 7 |
| Henan |  |  |  |  |  |  |  |  |  | 4 | 4 | 8 |
| Jilin |  |  |  |  |  |  |  |  |  | 4 |  | 4 |
| Jiangsu |  |  |  |  |  |  |  |  |  | 2 | 28 | 30 |
| Ningxia |  |  |  |  |  |  |  |  |  | 5 |  | 5 |
| Yunnan |  |  |  |  |  |  |  |  |  | 4 |  | 4 |
| Total | 3 | 4 | 2 | 4 | 14 | 11 | 2 | 3 | 67 | 126 | 90 | 326 |
